# Assessment of Functional and Radiological Outcomes of Comminuted Intra-Articular Distal Radius Fracture Treated With Locking Compression Plate

**DOI:** 10.7759/cureus.21398

**Published:** 2022-01-19

**Authors:** Shivam Patel, Ashwin Deshmukh, Parth Yadav, Mukesh Phalak, Sagar Gurnani, Siddharth Yadav, Anurag Anand

**Affiliations:** 1 Orthopaedics, Dr. D. Y. Patil Medical College, Hospital & Research Centre, Pune, IND; 2 Orthopaedics and Trauma, Dr. D. Y. Patil Medical College, Hospital & Research Centre, Pune, IND

**Keywords:** radiological outcome, locking compression plate, modified mayo score, functional outcome, distal end radius fracture

## Abstract

Background

Distal radial fractures (DRFs) are a prevalent form of skeletal injuries that hinder a person from performing daily living activities. Although several treatment modalities have been established to manage DRF, an optimal intervention has not been identified for comminuted fractures. The use of locking compression plates (LCPs) is gaining popularity for fractures that cannot be anatomically reduced because they offer better stability and early recovery. Thus, this study aims to investigate the surgical outcome of comminuted intra-articular DRFs treated with LCPs.

Methodology

We performed open reduction and internal fixation in 30 patients (18 males and 12 females) with DRF and were followed up at regular intervals following the surgery (at three, six, twelve, and twenty-four weeks). The surgical outcome was assessed both from functional and radiological standpoints. The influence of LCP on functional outcome was evaluated based on the modified Mayo wrist score and the quality of reduction based on the Lindstrom criteria by observing volar tilt and radial inclination.

Results

For radiological outcome, there was no variation in tilt/inclination of more than two degrees even after three months, which was a satisfactory result. Moreover, patients of all age groups showed early range of motion and functional benefit from LCP treatment. After six months of treatment, the patients experienced no pain and were able to return to their pre-injury jobs with little difficulty. Range of motion, work status, and grip strength after six months showed improvement of 15%, 7.8%, and 56%, respectively, compared to immediate postoperative assessments at three weeks. Although the end outcome with any treatment modality may yield similar results, when using LCPs, most patients experienced early functional improvement nearly six months after surgery.

Conclusions

By offering a lower risk of complications and early functional mobility, LCPs tend to restore the articular architecture of the fractured joint that results in the desired range of motion, grip strength, improved pain management, and functional status. Thus, LCPs appear to be a better alternative for distal end radial fractures than other treatment modalities.

## Introduction

Accounting for more than one-sixth of all fractures treated in the emergency room [1], distal radial fracture (DRF) is prevalently encountered in orthopedic practice [2]. DRF has a high incidence of 20-40 per 10,000 person-years [1,3]. Moreover, its incidence is similar among women and men below 50 years of age, and women over 80 years have the highest incidence of 120 per 10,000 person-years [1].

High-energy trauma (e.g., sports injuries, falls from height, and traffic accidents) is a common cause of fractures among adolescents and young adults; however, the elderly population experience osteoporotic low-energy fractures [4]. Fractures of distal radius typically occur after an impact on the outstretched hand, with the type of fracture depending upon the loading rate and the magnitude and direction of the load. Overall, 90% of the radius fractures are caused by stress loading with the wrist in dorsiflexion. The consequences of trauma to the hand severely impede a person from carrying out their activities of daily living; moreover, the treatment and management of DRF have been widely studied.

Management of DRF is debated as several factors such as fracture stability, displacement, patient characteristics, and activity demands can influence the type of treatment. Non-displaced fractures are managed non-operatively in a custom orthosis or OTC brace for four to six weeks, while displaced fractures are reduced and treated in the same manner. According to a systematic review of 37 studies, inadequate evidence exists to propose the best casting technique and the time of immobilization during the management of DRFs [5]. In an unstable fracture such as a comminuted fracture, operative intervention is preferred. Additionally, external fixation [6] or percutaneous pinning [7] is widely used. However, due to the complication of over-reduction, percutaneous pinning can only be an effective method for treating certain DRFs [8]. Moreover, treating DRFs with external fixation using the bridging technique does not always guarantee radial length [9]. External fixators can lead to stretching the wrist joint and shortening of the fracture if there is comminution or less than perfect apposition of the volar cortex. Thus, the use of external fixation in India has decreased during the last decade in favor of internal fixation [3]. Moreover, the previous two decades have seen gaining popularity [3,10] for open reduction and internal fixation with dorsal plates [11] or volar plates and stable angle screws [12].

Open reduction and internal fixation of DRFs are frequently used to manage unstable fractures. It includes volar plates, radial plates, dorsal plates, and fragment-specific fixation. Rikli and Regazzoni [11] introduced dorsal plates placed on the lateral and intermediate columns of the wrist. Later, the introduction of angular stable locking screws enabled volar fixation for dorsally displaced DRFs [12]. During the last decade, locking compression plate (LCP) has become common for surgical treatment in unstable DRFs [14]. Prospective evaluation has indicated that locked volar plating improves postoperative radiographs, strength, and range of motion (ROM) better than external fixation [15-17]. Using a volar locking plate also results in a faster recovery of function than percutaneous methods [16,17]. In a randomized study [18], palmar plating demonstrated significantly better results than dorsal plating when ROM, grip strength, and pain were evaluated. However, the Patient-Rated Wrist Evaluation (PRWE), Disabilities of the Arm, Shoulder and Hand (DASH), and quickDASH scores are significantly different after one year or more [15-17,19,20]. Similarly, the DASH score was significantly better for volar locked plates than external fixation in a meta-analysis. However, the difference was smaller than the minimal clinically significant difference estimated for the DASH score [21].

Although comminuted DRF can be treated with various methods, effective treatment management has not yet been identified. Moreover, fractures may displace later despite successful reduction due to comminution unless it is adequately managed because of the difficulty in evaluating the fracture stability and displacement. Additionally, the patient’s ability to carry out daily activities is also affected during recuperation. In this regard, LCP appears to be a viable treatment for comminuted DRF as it has fewer complications and tends to provide early recovery. Thus, to assess the viability of using LCPs to manage intra-articular distal end radius fractures, this study aims to prospectively evaluate the surgical outcome, both functional and radiological, at regular intervals.

## Materials and methods

A prospective observational study was conducted to assess the effect of LCP on the functional/radiological outcomes while managing comminuted intra-articular distal end radius fractures.

This study was conducted in the Department of Orthopaedics between May 2019 to May 2021. Ethical clearance was obtained from the Meeting of Research and Recognition Committee under the faculty of Medicine (IESC/PGS/2019/95). Informed consent was obtained from all participants before surgical procedures. Patients considered for the study underwent surgical procedures as per the standard guidelines. Pre and postoperative assessments were performed to evaluate their functional outcome. Finally, statistical analysis was performed to assess the influence of LCP before and after surgery.

Inclusion and exclusion criteria

Patients were included in the study if they were over 18 years of age; presented within two weeks of fracture, namely, displaced, comminuted, or closed fractures; and had intra-articular fractures (fracture with intra-articular displacement of 1 mm, 2 mm, or the degree of displacement) decided by X-ray.

Patients were excluded from the study if they were less than 18 years of age; had pathological, open, or undisplaced fractures; had pre-existing functional impairment of the ipsilateral upper limb; had neurological and psychiatric disorders that would preclude assessment; were considered unfit for surgery; and were unwilling to participate.

Surgical procedure

All surgical procedures were carried out by a team of two experienced (minimum of five years of experience) general orthopedic surgeons of the institute under a general or local anesthetic. As standard practice, we used preoperative prophylactic intravenous cefuroxime along with tourniquet and bipolar diathermy for homeostasis. An appropriate surgical strategy was adopted based on the fracture pattern. In addition, an image intensifier was used to evaluate fracture reduction and fixation process. Six types of variable-angle LCPs were used in the study, including the Small T locking plate along with the right and left side variants of distal radius volar locking plate, oblique locking plate, D/R extra-articular LCP (Head 5H), Distal radius styloid LCP, and D/R oblique articular LCP (Head 5H).

Evaluation of surgical outcome

Both functional and radiological assessments were performed at two weeks, six weeks, twelve weeks, and six months to evaluate the overall surgical outcome. Functional outcome was assessed using the modified Mayo wrist score. Factors such as pain, ROM, functional status (employment status), and grip strength were recorded at various time intervals to quantify the functional outcome. Postoperative radiographs were obtained within three days of surgery. The quality of reduction was classified based on Sarmiento’s modification of Lindstrom criteria. Volar tilt, ulnar variance, and radial inclination were radiographic parameters to assess DRF reduction.

Statistical analysis

Radiological and functional effects of LCP in the management of comminuted intra-articular distal end radius fractures were inferred from statistical analysis. Microsoft Excel was used for data entry, and analysis was performed using EPI-Info 7. Appropriate parametric/non-parametric tests were performed to determine statistical differences in functional outcomes by comparing the outcomes immediately after surgery and after two, six, twelve, and twenty-four weeks of recovery. Accordingly, the two groups were compared using the chi-square test/Fisher’s exact for categorical data and Student’s t-test/Mann-Whitney U test for continuous data.

## Results

Open reduction and internal fixation procedures were performed on 30 patients with DRF. The study included 18 male and 12 female patients aged 18 to 83 years. Patients were followed up at three, six, twelve, and twenty-four weeks and evaluated based on the assessment scores.

Radiological outcomes

**Figure 1:**
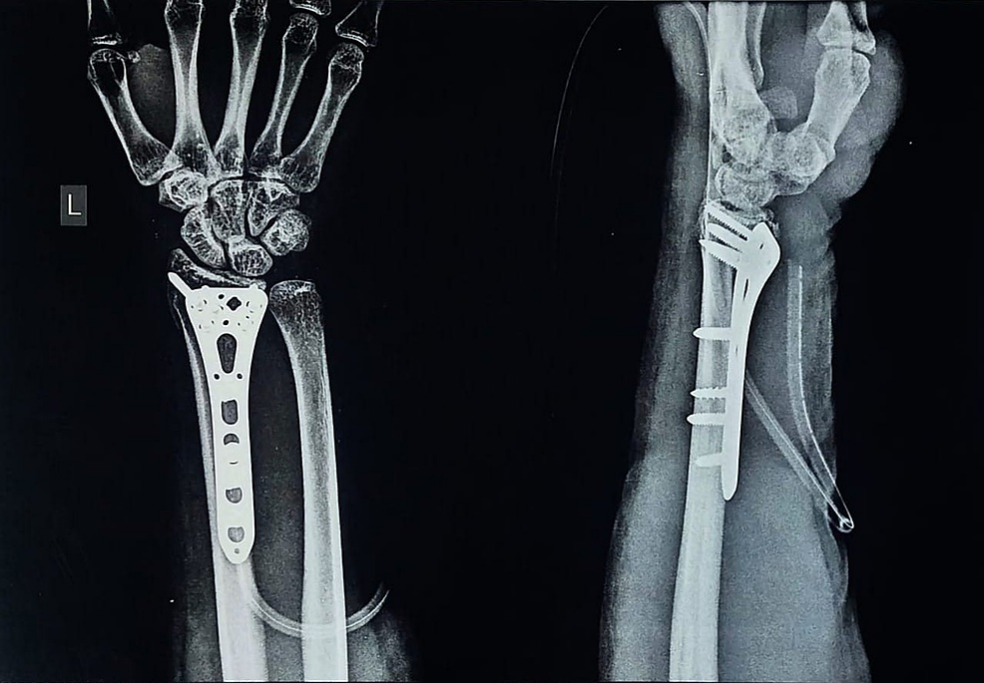
Postoperative X-ray of intra-articular distal end radius fracture treated with locking compression plate.

The radiological outcome was assessed based on volar tilt and radial inclination (Figure 1). The volar tilt and radial inclination following postoperative assessment were 9.3 ± 2.6 degrees (minimum: 2 degrees, maximum: 12 degrees) and 20.7 ± 2.1 degrees (minimum: 18 degrees, maximum: 26 degrees), respectively. Moreover, no change was observed in volar tilt (8.4 ± 2.4 degrees) and radial inclination (19.9 ± 2.4 degrees) between assessments after three weeks and three months following surgery.

Table 1 reports the change in volar tilt and radial inclination postoperatively and after three months. The average shift in volar tilt was 0.9 degrees. No change in volar tilt was larger than 2 degrees in any patient. Similar to volar tilt, the average shift in radial inclination was 0.8 degrees. Moreover, even after three months, all patients had no change in tilt/inclination of greater than 2 degrees. This was observed to be a satisfactory result. Moreover, on immediate postoperative radiological assessment (X-rays), there was no indication of articular steps (intra-articular step-off). This was the same during follow-up at three weeks and three months, and no significant change in radio-ulnar variance was observed.

**Table 1:** Changes in volar tilt and radial inclination postoperatively and after three months. P-value of ≤0.05 indicates a statistically significant difference between the groups compared. SD: standard deviation

Parameters	Time interval	Mean	SD	P-value
Volar tilt	Postoperative	9.3	2.6	<0.001
	At three months	8.4	2.4	
Radial inclination	Postoperative	20.7	2.1	<0.001
	At three months	19.9	2.4	

Functional outcomes

**Figure 2:**
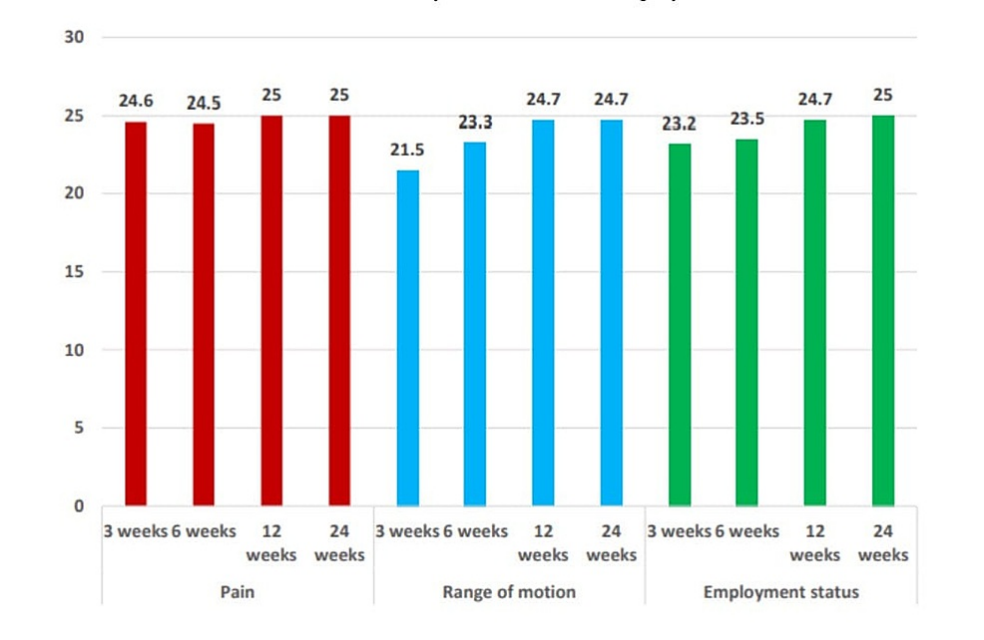
Assessment of pain, range of motion, and functional status at various time intervals.

Pain, ROM, and functional status at various time intervals were obtained to quantify the modified Mayo score. Assessments at three, six, twelve, and twenty-four weeks are shown in Figure 2. The pain, ROM, and functional status after three and twenty-four weeks of surgery are shown in Table 2.

**Table 2:** Change in pain, range of motion, and employment status after three and twenty-four weeks. P-value of ≤0.05 indicates a statistically significant difference between the groups compared. SD: standard deviation

Parameters	Time interval	Mean	SD	P-value
Pain	3 weeks	24.6	1.9	0.023
	24 weeks	25	0	
Range of motion	3 weeks	21.5	3.5	<0.001
	24 weeks	24.7	1.3	
Employment status	3 weeks	23.2	2.5	<0.001
	24 weeks	25	0	

**Figure 3:**
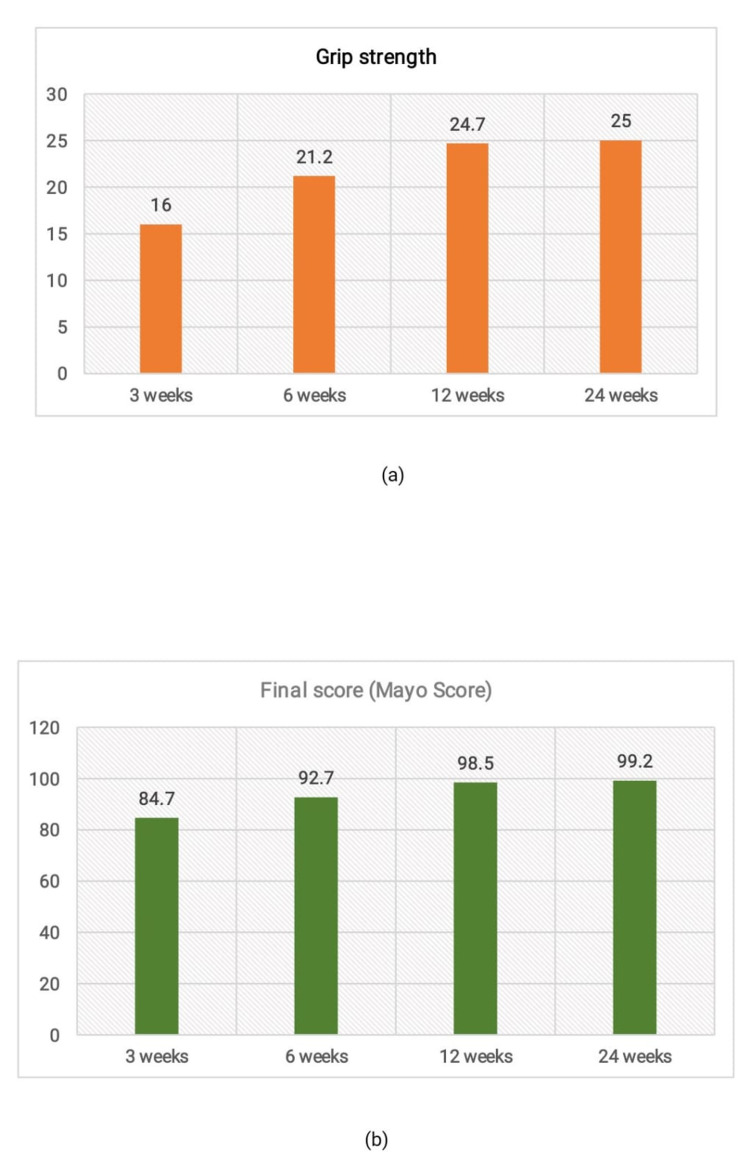
Assessment of (a) grip strength and (b) final Mayo score at various time intervals.

Assessments of grip strength and final Mayo score at three, six, twelve, and twenty-four weeks are shown in Figure 3a and Figure 3b, respectively. Table 3 shows the change in grip strength and final mayo score after three and twenty-four weeks of surgery. After three weeks, the average Mayo score was 24.6. With 25 patients having no pain, 13 patients had wrist and forearm movement similar to their opposite side, with an average ROM of 21.5. Moreover, 19 patients returned to their pre-injury employment status with little discomfort. The group average for employment status was 23.1. Lastly, five patients had grip strength greater than 90% when compared to the opposite side with an average Mayo score of 16.0 for grip strength. The score improved after six weeks, with 27 patients experiencing no pain; the average Mayo score of the group was 24.5. The average Mayo score for ROM was 23.3 in 21 patients who exhibited wrist and forearm movement similar to the opposite side. Twenty-one patients had difficulty returning to their pre-injury work status, with an average Mayo score of 23.5. When compared to the opposite side, 20 patients had grip strength greater than 90%, and their average Mayo score was 21.2.

**Table 3:** Changes in grip strength and final mayo score after three and twenty-four weeks. P-value of ≤0.05 indicates a statistically significant difference between the groups compared. SD: standard deviation

Parameters	Time interval	Mean	SD	P-value
Grip strength	3 weeks	16	44	<0.001
	24 weeks	25	0	
Final score (Mayo score)	3 weeks	84.7	9.3	<0.001
	24 weeks	99.2	1.9	

The score improved after 12 weeks, and all patients were pain-free, with an average Mayo score of 25 for the study group. In total, 28 patients had wrist and forearm movement similar to the opposite side, with an average Mayo score of 24.7 for ROM. With little discomfort, 28 patients returned to their pre-injury employment position, and their Mayo score for employment status was 24.7. When compared to the opposite side, 29 patients showed grip strength of >90%, and the average Mayo score for grip strength was 24.7.

**Figure 4:**
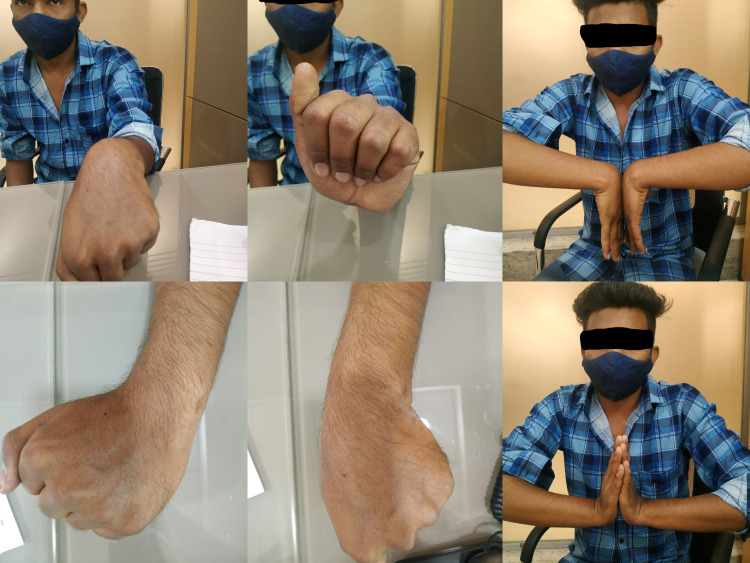
Different range of motion at wrist joint after six months of surgery.

The score improved after six months (Table 3). All patients had no pain, with an average Mayo score of 25. The average Mayo score for ROM was 24.7 in 28 patients who had the same wrist and forearm movement as the opposite side (Figure 4). All patients returned to their pre-injury jobs with little difficulty, and the average Mayo score for work status was 25. The average Mayo score for grip strength was 21.2, with all patients having grip strength greater than 90% compared to the other side.

At three weeks, type A/B fractures (modified AO classification) had good and excellent results, while type C fractures had satisfactory results. By six months, even satisfactory results had improved. Although 19 of the 30 patients possibly had weak bones due to old age, the surgery’s prognosis was unaffected. The seven older patients recovered quickly and had good functional mobility with few problems.

We faced some complications during our study. One of the patients who had good outcomes had a seizure, which led to poor postoperative rehabilitation. In another patient, a revision surgery had to be performed after a botched k-wiring effort four weeks before presentation. One patient developed a superficial infection that required debridement and three weeks of antibiotics. These patients had good outcomes by three months and great outcomes by six months. In three months, patients with superficial infections had good outcomes and excellent outcomes by six months. Apart from a few mild issues, no early or late consequences such as median nerve superficial nerve injury, arthritis, iatrogenic radial aneurysm, tendon rupture, or implant loosening were documented. For acute and unstable DRF, this study demonstrates open reduction with LCP as a safe and effective method of treatment. No other treatment method can provide adequate stability and allow early mobility.

## Discussion

The major goal of our study was to examine how well LCP worked for comminuted intra-articular distal end radius fractures. The use of LCP for distal end radius fractures has been shown to result in rapid movement and function recovery. Because LCP does not require immobilization, wrist movement can begin as early as the first postoperative day. Moreover, there is no loss in reduction, which allows for early return of ROM and power. Our results compare well with other known series of open reduction and fixation fracture therapy. Moreover, the early mobilization with LCP is not seen with any other technique.

The use of LCP for DRFs resulted in wrist mobilization as early as the first postoperative day, allowing the majority of our patients to resume their normal activities the next day after surgery. The added benefits included the improvement in their quality of life and prompt return to their daily routines. From the first postoperative day, our patients were able to perform most activities of daily living such as eating, tooth brushing, reading, and writing. There are no other therapy options that provide this level of early functional mobility. K-wire stabilization, buttress plating, closed reduction and casting, and external fixation, for example, require a minimum of six weeks of immobilization followed by a varying duration of physiotherapy to return to the functional condition prior to the injury.

The Mayo scores recorded at three weeks demonstrated the superiority of LCP over other treatment approaches. Patients of all age groups showed an early range of mobility and function and benefited from LCP treatment. In addition, early recovery was linked to a low risk of complication, which has been reported in other studies. For quick recovery, patients should adhere to vigorous physiotherapy treatments. One of the primary reasons for the quick recovery of patients was a focus on rigorous and supervised physiotherapy and correct reduction and solid fixation achieved by locking plates. To further confirm this finding, studies focusing on the importance of early, aggressive, and supervised physiotherapy for rapid recovery of wrist functions following a distal end radius fracture are needed.

Simple undisplaced fractures produced good results, while complex comminuted displaced fractures produced satisfactory results. This may lead to the conclusion that the more complicated the fracture pattern, the longer the recuperation period. Although the outcome with any treatment modality may yield similar results, when using LCPs, most patients experienced early functional improvement nearly six months after surgery compared to one year in other treatment modalities.

Treatment with LCP was also found to be beneficial for elderly patients with osteoporosis, with early improvements in mobility and function. Moreover, early recovery was linked to a low risk of complication, as shown in other studies. In our view, restoring the articular architecture of the joint resulted in the desired ROM, grip strength, pain severity, and functional status. Hence, compared to other current modalities, it seems reasonable to employ LCPs for distal end radius fractures as a successful treatment modality for early functional mobility. When compared to other methods, the use of LCPs has a lower risk of complications.

Several limitations of our research must be addressed. The number of cases included in the study and the duration of the follow-up were insufficient. We did not compare non-operative treatment and volar locking plate fixation. To assess the clinical outcome, further prospective trials with a large population and a longer follow-up period are required.

## Conclusions

We can obtain early functional mobility and reduce complications using LCPs. The use of LCPs allowed for early wrist mobilization as early as the first postoperative day. Most patients could resume their normal activities as soon as their postoperative discharge. This treatment modality allows patients to have a better quality of life and to return to work sooner. Because screw plate construction is of the locking type, it produces fixation in any bone type, including those with defects or osteoporosis.

## References

[1] Brogren E, Petranek M, Atroshi I (2007). Incidence and characteristics of distal radius fractures in a southern Swedish region. BMC Musculoskelet Disord.

[2] van Staa TP, Dennison EM, Leufkens HG, Cooper C (2001). Epidemiology of fractures in England and Wales. Bone.

[3] Mellstrand-Navarro C, Pettersson HJ, Tornqvist H, Ponzer S (2014). The operative treatment of fractures of the distal radius is increasing: results from a nationwide Swedish study. Bone Joint J.

[4] Lindau TR, Aspenberg P, Arner M, Redlundh-Johnell I, Hagberg L (1999). Fractures of the distal forearm in young adults. An epidemiologic description of 341 patients. Acta Orthop Scand.

[5] Handoll HH, Madhok R (2003). Conservative interventions for treating distal radial fractures in adults. Cochrane Database Syst Rev.

[6] Cooney WP (1983). External fixation of distal radial fractures. Clin Orthop Relat Res.

[7] Kapandji A (1987). [Intra-focal pinning of fractures of the distal end of the radius 10 years later]. Ann Chir Main.

[8] Gofton W, Liew A (2007). Distal radius fractures: nonoperative and percutaneous pinning treatment options. Orthop Clin North Am.

[9] McQueen MM (1998). Redisplaced unstable fractures of the distal radius. A randomised, prospective study of bridging versus non-bridging external fixation. J Bone Joint Surg Br.

[10] Wilcke MK, Hammarberg H, Adolphson PY (2013). Epidemiology and changed surgical treatment methods for fractures of the distal radius: a registry analysis of 42,583 patients in Stockholm County, Sweden, 2004-2010. Acta Orthop.

[11] Rikli DA, Regazzoni P (1996). Fractures of the distal end of the radius treated by internal fixation and early function. A preliminary report of 20 cases. J Bone Joint Surg Br.

[12] Orbay JL, Fernandez DL (2002). Volar fixation for dorsally displaced fractures of the distal radius: a preliminary report. J Hand Surg Am.

[13] Koval K, Haidukewych GJ, Service B, Zirgibel BJ (2014). Controversies in the management of distal radius fractures. J Am Acad Orthop Surg.

[14] Handoll HH, Madhok R (2003). Surgical interventions for treating distal radial fractures in adults. Cochrane Database Syst Rev.

[15] Mackenney PJ, McQueen MM, Elton R (2006). Prediction of instability in distal radial fractures. J Bone Joint Surg Am.

[16] Wright TW, Horodyski M, Smith DW (2005). Functional outcome of unstable distal radius fractures: ORIF with a volar fixed-angle tine plate versus external fixation. J Hand Surg Am.

[17] Wilcke MK, Abbaszadegan H, Adolphson PY (2011). Wrist function recovers more rapidly after volar locked plating than after external fixation but the outcomes are similar after 1 year. Acta Orthop.

[18] Karantana A, Downing ND, Forward DP (2013). Surgical treatment of distal radial fractures with a volar locking plate versus conventional percutaneous methods: a randomized controlled trial. J Bone Joint Surg Am.

[19] Jakubietz RG, Gruenert JG, Kloss DF, Schindele S, Jakubietz MG (2008). A randomised clinical study comparing palmar and dorsal fixed-angle plates for the internal fixation of AO C-type fractures of the distal radius in the elderly. J Hand Surg Eur Vol.

[20] Mellstrand Navarro C, Ahrengart L, Törnqvist H, Ponzer S (2016). Volar locking plate or external fixation with optional addition of K-wires for dorsally displaced distal radius fractures: a randomized controlled study. J Orthop Trauma.

[21] Walenkamp MM, Bentohami A, Beerekamp MS, Peters RW, van der Heiden R, Goslings JC, Schep NW (2013). Functional outcome in patients with unstable distal radius fractures, volar locking plate versus external fixation: a meta-analysis. Strategies Trauma Limb Reconstr.

